# Supplementary material for: Interoceptive awareness and beliefs about health and the body as predictors of the intensity of emotions experienced at the beginning of the pandemic
Source: PeerJ. 2021 Dec 21;9:e12542. doi: 10.7717/peerj.12542 (PMC8706328; doi:10.7717/peerj.12542)
Supplement: Supplemental Information 2 [file peerj-09-12542-s002.docx]

Appendix

**1. The sample characteristic**

Table A1

*Participant demographics*

| **Place of living** | **village** | **city with less than 50,000 inhabitants** | **city with more than 50,000 and less than 500,000 inhabitants** | **city with more than 500,000 and less than 1mln inhabitants** | **city with more than 1mln inhabitants** |
| --- | --- | --- | --- | --- | --- |
|  | 13,4% | 14,7% | 19,4% | 7,7% | 44,8% |
| **Educational background** | Less than High School | High School or equivalent | Higher degree - bachelor or more |  |  |
|  | 0,3% | 31,1% | 68,8% |  |  |
| **Having a stable source of income** | yes | No |  |  |  |
|  | 67,6% | 32,4% |  |  |  |
| **Evaluation of financial situation** | very bad | Bad | Moderate | good | very good |
|  | 0,7% | 5,4% | 34,8% | 40,1% | 19,1% |
| **Chronic disease** | yes | No |  |  |  |
|  | 30,% | 69,9% |  |  |  |
| **Knowing someone who had diagnosed COVID-19** | yes | No |  |  |  |
|  | 16,7% | 83,3% |  |  |  |
| **Developing COVID-19** | yes | No |  |  |  |
|  | 1% | 99% |  |  |  |

**2. Demographic data and different types of emotions**

**2.1. Demographic data and negative automatic and reflective emotions**

There is difference between women and men in the case of automatic negative emotions: women (*M* = 3.68; *SD* = 1.49); men (*M* = 2.96; *SD* = 1.51; *t* = 3.35; *p* = .001). At the same time, individuals with a stable source of income (*M* = 3.39; *SD* = 1.49) experience fewer negative automatic emotions compared to those without a stable source of income (*M* = 3.83; *SD* = 1.57; *t* = 2.37; *p* = 0.02). A similar effect was observed as a correlation between negative automatic emotions and perceived financial status (*r* = -.18 *p* = .001). There was also observed a low negative correlation with place of living (*r* = -.12 *p* = .038). Similarly, as in the previous analysis, people who suffer from chronic disease experience more negative automatic emotions (*M* = 3.81; *SD* = 1.56) than people who do not have such a problem (*M* = 3.42; *SD* = 1.50; *t* = 2.06; *p* = .04).

As mentioned previously, individuals with a stable source of income (*M* = 2.73; *SD* = 1.18) experience fewer negative reflective emotions compared to individuals without a stable source of income (*M* = 3.29; *SD* = 1.33; *t* =3.69; *p* < .001). There was also a negative low correlation for perceived financial status and negative reflective emotions (*r* = 0.24; *p* < .001). The better status is, the lower negative emotions are. No other statistically significant correlations were observed.

**2.2. Demographic data and positive automatic and reflective emotions**

Positive automatic emotions were related lowly and positively with perceived financial status (*r* = .12; *p* = .046). Additionally, women experience fewer positive automatic emotions (*M* = 2.94; *SD* = 1.21) in comparison to men (*M* = 3.28; *SD* = 1.13; *t* = -2.15, *p* = .033).

Positive reflective emotions were associated with age (*r* = .25; *p* < .001) and perceived financial status (*r* = -.18; *p* = .008). Moreover, individuals having a stable source of income experience more positive reflective emotions (*M* = 4.34; *SD* = 1.05) than those who do not (*M* = 3.79; *SD* = 1.19; *t* =-4.06, *p* < .001).

**3. Multidimensional interoceptive awareness, beliefs about health and body, emotions – bivariate correlations**

A correlation analysis was performed for the following pairs of variables: all types of emotions and MAIA’s scale, all types of emotions and negative beliefs about health and body, MAIA’s scales

Table A2

*Values of correlation coefficients.*

|  | Relationships | | | *r* | *p* |
| --- | --- | --- | --- | --- | --- |
|  | Emo. Neg. Aut. | <--> | Not-Worrying | -.31 | .001* |
|  | Emo. Neg. Aut. | <--> | Attention Reg. | -.13 | .021 |
|  | Emo. Neg. Aut. | <--> | Self-Regulation | -.24 | .001* |
|  | Emo. Neg. Aut. | <--> | Trusting | -.26 | .001* |
|  | Emo. Neg. Aut. | <--> | Noticing | .19 | .027 |
|  | Emo. Neg. Aut. | <--> | Emotional Awer. | .13 | .024 |
|  | Emo. Neg. Refl. | <--> | Not-Worrying | -.31 | .001* |
|  | Emo. Neg. Refl. | <--> | Attention Reg. | -.19 | .001* |
|  | Emo. Neg. Refl. | <--> | Self-Regulation | -.30 | .001* |
|  | Emo. Neg. Refl. | <--> | Trusting | -.32 | .001* |
|  | Emo. Pos. Aut. | <--> | Attention Reg. | .22 | .001* |
|  | Emo. Pos. Aut. | <--> | Self-Regulation | .31 | .001* |
|  | Emo. Pos. Aut. | <--> | Body Listening | .15 | .01 |
|  | Emo. Pos. Aut. | <--> | Trusting | .33 | .001* |
|  | Emo. Pos. Refl. | <--> | Not-Distracting | -.13 | .025 |
|  | Emo. Pos. Refl. | <--> | Attention Reg. | .22 | .001* |
|  | Emo. Pos. Refl. | <--> | Self-Regulation | .25 | .001* |
|  | Emo. Pos. Refl. | <--> | Trusting | .24 | .001* |
|  | Neg. Beliefs | <--> | Emo. Neg. Aut. | .42 | .001* |
|  | Neg. Beliefs | <--> | Emo. Neg. Refl. | .40 | .001* |
|  | Neg. Beliefs | <--> | Emo. Pos. Aut. | -.20 | .001* |
|  | Neg. Beliefs | <--> | Not-Worrying | -.32 | .001* |
|  | Neg. Beliefs | <--> | Trusting | -.11 | .027 |
|  | Neg. Beliefs | <--> | Noticing | .19 | .001* |
|  | Neg. Beliefs | <--> | Emotional Awar. | .17 | .003 |

*r* – correlation coefficient; *p* – statistical significance, after the correction for multiple comparison significance value: p < 0.002, significant values were marked with (*) symbol

**4. Variables predicting intensity of positive and negative emotions**

In the next step, models explaining emotions were tested in multiple block regression models.

**4.1. Negative automatic emotions**

Subsequently, the regression analysis was applied for automatic negative emotions as an explained variable. In the first step, demographic variables were entered into the model. Perceived financial status (Beta = -.15; *t* = -2.71; *p* = .007), gender (Beta = -.17; *t* = -3.08; *p* = .002), place of living (Beta = -.12; *t* = -2.16; *p* = .032) and suffering from chronic disease (Beta = -.12; *t* = -2.07; *p* = .040) were significant predictors (*F*(5, 293) = 6.53, *p* < .001, R-squared = .10). Having a stable source of income variable was not significant. In the second step, negative beliefs about health and the body (Beta = .39; *t* = 6.83; *p* < .001) were included in the model, which improved the model adjustment, delta-F(1, 293) = 46.63, *p* < .001, delta-R-squared = .13.. Gender and suffering from chronic disease turned out to be insignificant. Finally, the MAIA questionnaire scales were added to the model: Not-Worrying (Beta = -.15; *t* = -2.67; *p* = .008); Self-Regulation (Beta = -.23; *t* = -3.41; *p* = .001) and Trusting (Beta = -.13; *t* = -2.21; *p* = .028), delta-F(6, 289) = 6.07, *p* < .001, delta-R-squared = .09. After the back elimination of the weakest insignificant predictors, the final model was built and it included the following predictors: perceived financial status (Beta = -.11; *t* = -2.07; *p* = .039), negative beliefs about health and the body (Beta = .33; *t* = 6.04; *p* < .001) and three scales of the MAIA questionnaire: Not-Worrying (Beta = -.16; *t* = -2.90; *p* = .004), Self-Regulation (Beta = -.12; *t* = -2.09; *p* = 0.037) and Trusting (Beta = -.12; *t* = -2.09; *p* = .038). The model showed good adjustment to data *F*(5, 293) = 21.21, *p* < .001, R-squared = .25.

**4.2. Negative reflective emotions**

Subsequently, the model for reflective negative emotions as the explained variable was run. In the first step, a demographic variables were entered into the model *F*(2,296) = 14.02, p < .001, R-squared = .08. Two of them turned out to be significant: perceived financial status (Beta = -.21; *t* = -3.63; *p* < .001) and a stable source of income (Beta = -.16; *t* = -2.78; *p* = .006). In the next step, negative beliefs about health and the body (Beta = .38; *t* = 7.35; *p* < .001) were included in the model, delta-F(1, 295) = 54.08, *p* < .001, delta-R-squared = .14. Then, interoceptive awareness scales were included in the model F(4, 291) = 11.48, *p* < .001, R-squared = .11. Not-Worrying (Beta = -.16; *t* = -3.06; *p* = .002), Self-Regulation (Beta = -.21; *t* = -3.23; *p* = .001) and Trusting (Beta = -.18; *t* = -3.05; *p* = .002) were statistically significant. The final model included perceived financial status (Beta = -0.13; *t* = -2.58; *p =* .010) and having a stable source of income (Beta = -0.18; *t* = -3.64; p < .001) as controlled demographic variables. Other predictors were negative beliefs about health and the body (Beta = .30; *t* = 5.78; *p* < .001) and interoceptive awareness scales: Not-Worrying (Beta = -.15; *t* = -2.82; *p* = .005), Self-Regulation (Beta = -.16; *t* = -2.83; *p* = .005) and Trusting (Beta = -.15; t = -2.73; *p* = .006). The model revealed a good adjustment to data *F*(6, 292) = 23.73, *p* < .001, R-squared = 0.33.

**4.3. Positive automatic emotions**

In the case of positive automatic emotions, in the first step, perceived financial status and gender were entered into the model *F*(2, 296) = 3.57, *p* = .03, R-squared = 0.02; only perceived financial status was significant (Beta = .12; *t* = 2.02; *p* = .044). In the next step, the variable of negative beliefs about health and the body (Beta = -.17; *t* = -2.86; *p* = .004) was added to the model, delta-F(1, 295) = 8.20, *p* = .001, delta-R-squared = .04. Perceived financial status became insignificant. Then the MAIA questionnaire scales were included, two of which were statistically significant: Self-Regulation (Beta = .25; *t* = 3.26; *p* = .001) and Trusting (Beta = .26; *t* = 3.92; *p* < .001), delta-F(4, 293) = 11.23, *p* < .001, delta-R-squared = .11. The final model included three predictors: negative beliefs about health and the body (Beta = -.15; *t* = -2.71; *p* = .007); Self-Regulation (Beta = 0.19; *t* = 3.11; *p* = .002); and Trusting (Beta = 0.22; *t* = 3.52; *p* < .001). They explained about 16% of the explained variables’ variance, *F*(3, 295) = 18.73, *p* < .001, R-squared = .16.

**4.4. Positive reflective emotions**

Subsequently, the model for positive reflective emotions was run. In the first step, demographic variables were included: age (Beta = .21; *t* = 3.63; *p* = .001); having a stable source of income (Beta = .14; *t* = 2.45; *p* = .015); and perceived financial status (Beta = .12; *t* = 2.18; *p =* .030). The first model explained about 10% of positive automatic emotion variance *F*(3, 295) = 11.38, *p* < .001, R-squared = .10. In the next step, negative beliefs about health and the body were added; however, the change in the model was not significant.. Finally, four scales of the MAIA questionnaire were included in the model, delta-F(4, 291) = 7.10, *p* < .001, delta-R-squared = .08; none of them were statistically significant. After back reduction of insignificant predictors, the final model was developed. The final model included: age (Beta = 0.19; *t* = 3.23; *p* = .001); having a stable source of income (Beta = 0.17; *t* = 3.01; *p* = .003); and Self-Regulation (Beta = 0.22; *t* = 4.11; *p* < .001). The model revealed a good adjustment to data *F*(3, 295) = 15.82, *p* < .001, R-squared = .13.

The summary of explanatory models for all types of emotions can be found in the table below.

Table A3

*Negative and positive automatic and reflective emotion predictors.*

| Explained variable / Predictors | β | T | P | F | P | R^2^ |
| --- | --- | --- | --- | --- | --- | --- |
| Negative automatic emotions |  |  |  | 21.21 | < .001 | .25 |
| Perceived financial status | -.11 | -2.07 | = .039 |  |  |  |
| Negative beliefs 1 | .33 | 6.04 | < .001 |  |  |  |
| Not-Worrying | -.17 | -2.90 | = .004 |  |  |  |
| Self-Regulation | -.12 | -2.09 | = .037 |  |  |  |
| Trusting | -.12 | -2.09 | =.038 |  |  |  |
| Negative reflective emotions |  |  |  | 23.73 | < .001 | .33 |
| Perceived financial status | -.13 | -2.58 | = .010 |  |  |  |
| Stable source of income | -.18 | -3.64 | < .001 |  |  |  |
| Negative beliefs 1 | .30 | 5.78 | < .001 |  |  |  |
| Not-Worrying | -.15 | -2.82 | = .005 |  |  |  |
| Self-Regulation | -.16 | -2.83 | = .005 |  |  |  |
| Trusting | -.15 | -2.73 | = .006 |  |  |  |
| Positive automatic emotions |  |  |  | 18.73 | < .001 | .16 |
| Negative beliefs 1 | -.15 | -2.71 | = .007 |  |  |  |
| Self-Regulation | .19 | 3.11 | = .002 |  |  |  |
| Trusting | .21 | 3.52 | < .001 |  |  |  |
| Positive reflective emotions |  |  |  | 15.83 | < .001 | .13 |
| Age | .19 | 3.23 | = .001 |  |  |  |
| Stable source of income | .17 | 3.01 | = .003 |  |  |  |
| Self-Regulation | .22 | 4.11 | < .001 |  |  |  |
