# Supplementary material for: Interoceptive awareness and beliefs about health and the body as predictors of the intensity of emotions experienced at the beginning of the pandemic
Source: PeerJ. 2021 Dec 21;9:e12542. doi: 10.7717/peerj.12542 (PMC8706328; doi:10.7717/peerj.12542)
Supplement: Supplemental Information 3 — In the study, the polish adaptation of the questionnaire by Anna Kozieł, Anna Brytek-Matera was used. [file peerj-09-12542-s003.pdf]

Multidimensional Assessment of Interoceptive Awareness (MAIA)

Contact: Wolf E. Mehling, MD
Osher Center for Integrative Medicine
University of California, San Francisco
1545 Divisadero St., 4th floor
San Francisco, CA 94115
Phone: 01 (415) 353 9506
mehlingw@ocim.ucsf.edu
http://www.osher.ucsf.edu/maia/

MAIA ©2012, University of California San Francisco
http://www.osher.ucsf.edu/maia/

Multidimensional Assessment of Interoceptive Awareness

Permission and Copyright

Although the MAIA survey is copyrighted, it is available without charge and no written permission is required for its use. This assumes agreement with the following as a consequence of using a MAIA survey:

- Please refer to the survey using its complete name – Multidimensional Assessment of Interoceptive Awareness - and provide the appropriate citation.
- Modifications may be made without our written permission. However, please clearly identify any modifications in any publications as having been made by the users. If you modify the survey, please let us know for our records.
- We recommend including entire subscales when selecting items from the MAIA to retain the psychometric features of these subscales (rather than selecting items from subscales).
- If you translate the MAIA into another language, please send us a copy for our records.
- If other investigators are interested in obtaining the survey, please refer them to the source document (PLoS-ONE 2012, and www.osher.ucsf.edu/maia/) to assure they obtain the most recent version and scoring instructions.

Scoring Instructions

Take the average of the items on each scale.
Note: Reverse-score items 5, 6, and 7 on Not-Distracting, and items 8 and 9 on Not-Worrying.

1. **Noticing:** Awareness of uncomfortable, comfortable, and neutral body sensations

Q1______ + Q2______ + Q3______ + Q4______ / 4 = ___________

2. **Not-Distracting:** Tendency not to ignore or distract oneself from sensations of pain or discomfort

Q5(**reverse**)______ + Q6(**reverse**)______ + Q7(**reverse**)______ / 3 = ___________

3. **Not-Worrying:** Tendency not to worry or experience emotional distress with sensations of pain or discomfort

Q8(**reverse**)______ + Q9(**reverse**)______ + Q10______ / 3 = ___________

4. **Attention Regulation:** Ability to sustain and control attention to body sensations

Q11_____ + Q12_____ + Q13_____ + Q14_____ + Q15_____ + Q16_____ + Q17_____ / 7 = ________

5. **Emotional Awareness:** Awareness of the connection between body sensations and emotional states

Q18_____ + Q19_____ + Q20_____ + Q21_____ + Q22_____ / 5 = ___________

6. **Self-Regulation:** Ability to regulate distress by attention to body sensations

Q23_____ + Q24_____ + Q25_____ + Q26_____ / 4= ___________

7. **Body Listening:** Active listening to the body for insight

Q27_____ + Q28_____ + Q29_____ / 3= ___________

8. **Trusting:** Experience of one's body as safe and trustworthy

Q30_____ + Q31_____ + Q32_____ / 3= ___________

Below you will find a list of statements. Please indicate how often each statement applies to you generally in daily life.

.	Circle one number on each line					
	**Never**					**Always**
1. When I am tense I notice where the tension is located in my body.	0	1	2	3	4	5
2. I notice when I am uncomfortable in my body.	0	1	2	3	4	5
3. I notice where in my body I am comfortable.	0	1	2	3	4	5
4. I notice changes in my breathing, such as whether it slows down or speeds up.	0	1	2	3	4	5
5. I do not notice (I ignore) physical tension or discomfort until they become more severe.	0	1	2	3	4	5
6. I distract myself from sensations of discomfort.	0	1	2	3	4	5
7. When I feel pain or discomfort, I try to power through it.	0	1	2	3	4	5
8. When I feel physical pain, I become upset.	0	1	2	3	4	5
9. I start to worry that something is wrong if I feel any discomfort.	0	1	2	3	4	5
10. I can notice an unpleasant body sensation without worrying about it.	0	1	2	3	4	5
11. I can pay attention to my breath without being distracted by things happening around me.	0	1	2	3	4	5
12. I can maintain awareness of my inner bodily sensations even when there is a lot going on around me.	0	1	2	3	4	5
13. When I am in conversation with someone, I can pay attention to my posture.	0	1	2	3	4	5
14. I can return awareness to my body if I am distracted.	0	1	2	3	4	5
15. I can refocus my attention from thinking to sensing my body.	0	1	2	3	4	5
16. I can maintain awareness of my whole body even when a part of me is in pain or discomfort.	0	1	2	3	4	5

MAIA ©2012, University of California San Francisco
http://www.osher.ucsf.edu/maia/

Please indicate how often each statement applies to you generally in daily life.

	Circle one number on each line					
	Never					Always
17. I am able to consciously focus on my body as a whole.	0	1	2	3	4	5
18. I notice how my body changes when I am angry.	0	1	2	3	4	5
19. When something is wrong in my life I can feel it in my body.	0	1	2	3	4	5
20. I notice that my body feels different after a peaceful experience.	0	1	2	3	4	5
21. I notice that my breathing becomes free and easy when I feel comfortable.	0	1	2	3	4	5
22. I notice how my body changes when I feel happy / joyful.	0	1	2	3	4	5
23. When I feel overwhelmed I can find a calm place inside.	0	1	2	3	4	5
24. When I bring awareness to my body I feel a sense of calm.	0	1	2	3	4	5
25. I can use my breath to reduce tension.	0	1	2	3	4	5
26. When I am caught up in thoughts, I can calm my mind by focusing on my body/breathing.	0	1	2	3	4	5
27. I listen for information from my body about my emotional state.	0	1	2	3	4	5
28. When I am upset, I take time to explore how my body feels.	0	1	2	3	4	5
29. I listen to my body to inform me about what to do.	0	1	2	3	4	5
30. I am at home in my body.	0	1	2	3	4	5
31. I feel my body is a safe place.	0	1	2	3	4	5
32. I trust my body sensations.	0	1	2	3	4	5

MAIA ©2012, University of California San Francisco
http://www.osher.ucsf.edu/maia/
