# Supplementary material for: Interoceptive awareness and beliefs about health and the body as predictors of the intensity of emotions experienced at the beginning of the pandemic
Source: PeerJ. 2021 Dec 21;9:e12542. doi: 10.7717/peerj.12542 (PMC8706328; doi:10.7717/peerj.12542)
Supplement: Supplemental Information 4 [file peerj-09-12542-s004.docx]

**The set of questionnaires used on Qualtrics platform – English translation:**

1. Assessment of currently experienced emotions divided based on valence and origin.
2. Multidimensional Assessment of Interoceptive Awareness (MAIA) Wolf E. Mehling, in polish adaptation of Anna Kozieł, Anna Brytek-Matera

The original English version of the tool is available under the link: <https://osher.ucsf.edu/sites/osher.ucsf.edu/files/inline-files/maia1.pdf>

1. The list of negative beliefs about health and body.
2. Demographic questions.
3. Assessment of currently experienced emotions divided based on valence and origin.

Assessment of experienced emotions

Please mark on the scale below to what extent you are experiencing these emotions now.

1 - means "to a small extent"

7 - means "to a significant extent"

- Suffering 1 ----- 2 ----- 3 ----- 4 ----- 5 ----- 6 ----- 7
- Helplessness 1 ----- 2 ----- 3 ----- 4 ----- 5 ----- 6 ----- 7
- Frustration 1 ----- 2 ----- 3 ----- 4 ----- 5 ----- 6 ----- 7
- Breakdown 1 ----- 2 ----- 3 ----- 4 ----- 5 ----- 6 ----- 7
- Terror 1 ----- 2 ----- 3 ----- 4 ----- 5 ----- 6 ----- 7
- Shame 1 ----- 2 ----- 3 ----- 4 ----- 5 ----- 6 ----- 7
- Sadness 1 ----- 2 ----- 3 ----- 4 ----- 5 ----- 6 ----- 7
- Dejection 1 ----- 2 ----- 3 ----- 4 ----- 5 ----- 6 ----- 7
- Depression 1 ----- 2 ----- 3 ----- 4 ----- 5 ----- 6 ----- 7
- Envy 1 ----- 2 ----- 3 ----- 4 ----- 5 ----- 6 ----- 7
- Alleviation 1 ----- 2 ----- 3 ----- 4 ----- 5 ----- 6 ----- 7
- Reassurance 1 ----- 2 ----- 3 ----- 4 ----- 5 ----- 6 ----- 7
- Excitation 1 ----- 2 ----- 3 ----- 4 ----- 5 ----- 6 ----- 7
- Relaxation 1 ----- 2 ----- 3 ----- 4 ----- 5 ----- 6 ----- 7
- Tenderness 1 ----- 2 ----- 3 ----- 4 ----- 5 ----- 6 ----- 7
- Persistence 1 ----- 2 ----- 3 ----- 4 ----- 5 ----- 6 ----- 7
- Compassion 1 ----- 2 ----- 3 ----- 4 ----- 5 ----- 6 ----- 7
- Hope 1 ----- 2 ----- 3 ----- 4 ----- 5 ----- 6 ----- 7
- Courage 1 ----- 2 ----- 3 ----- 4 ----- 5 ----- 6 ----- 7
- Loyalty 1 ----- 2 ----- 3 ----- 4 ----- 5 ----- 6 ----- 7

1. Multidimensional Assessment of Interoceptive Awareness (MAIA)

Source: <https://osher.ucsf.edu/sites/osher.ucsf.edu/files/inline-files/maia1.pdf> (access from 29th of December 2020)

The pdf file was added as supplemental file.

1. The list of negative beliefs about health and body.

Reaction to the Current Coronavirus Epidemic - Beliefs About Health and Body

Statements about different health beliefs in connection with the current Coronavirus epidemic will be presented below. Please respond to each of them using a five-point scale of answers, where:

1 - means "I don't agree at all"

5 - means "I completely agree"

1. I believe that my health is in danger.

1 ----- 2 ----- 3 ----- 4 ----- 5

1. I believe that my life is in danger.

1 ----- 2 ----- 3 ----- 4 ----- 5

1. My immune system is not durable enough, to deal with the disease.

1 ----- 2 ----- 3 ----- 4 ----- 5

1. My body is weak.

1 ----- 2 ----- 3 ----- 4 ----- 5

1. I’m easily infected.

1 ----- 2 ----- 3 ----- 4 ----- 5

1. To detect potential symptoms as quickly as possible, you need to carefully monitor your body's functioning.

1 ----- 2 ----- 3 ----- 4 ----- 5

1. Even a small change in the functioning of my body can be a symptom of a developing disease.

1 ----- 2 ----- 3 ----- 4 ----- 5

1. I can't do anything to protect my body from getting sick.

1 ----- 2 ----- 3 ----- 4 ----- 5

1. My respiratory system is not as efficient as for other people’s respiratory systems.

1 ----- 2 ----- 3 ----- 4 ----- 5

1. If I fall ill, my body will definitely not be able to handle it.
2. ----- 2 ----- 3 ----- 4 ----- 5
3. Demographic questions.

DEMOGRAPHIC QUESTIONNAIRE

This survey asks you about your general current life situation. Please answer all the questions contained in it by choosing one of the answers.

1. Age:
2. Gender:

- man
- woman
- other

1. Place of living:

- village
- city below 50,000 residents
- city 50 - 500 thousand residents
- city of 500,000 - 1 million inhabitants
- a city with over 1 million inhabitants

1. Education

- none
- elementary school
- junior high school
- secondary education
- higher education

1. Do you have a stable source of income?

- yes
- no

1. How do you assess your financial situation?

- very good
- good
- moderate
- bad
- very bad

1. Do you suffer from chronic diseases?

- yes
- no

1. Do you know anyone who has fallen ill with the disease caused by the COVID-19 Coronavirus?

- yes
- no

1. Have you ever had a disease caused by the COVID-19 Coronavirus?

- yes
- no
